# Auditory and Visual Integration for Emotion Recognition and Compensation for Degraded Signals are Preserved With Age

**DOI:** 10.1177/23312165211045306

**Published:** 2021-10-07

**Authors:** Minke J. de Boer, Tim Jürgens, Deniz Başkent, Frans W. Cornelissen

**Affiliations:** 1 Research School of Behavioural and Cognitive Neuroscience, University of Groningen, Groningen, the Netherlands; 2 Department of Otorhinolaryngology, 10173 University Medical Center Groningen, University of Groningen, Groningen, the Netherlands; 3 Laboratory of Experimental Ophthalmology, 10173 University Medical Center Groningen, University of Groningen, Groningen, the Netherlands; 4 Institute of Acoustics, Technische Hochschule Lübeck, Lübeck, Germany; * These authors contributed equally to this work.

**Keywords:** aging, eye-tracking, audiovisual, emotion recognition, sensory impairments

## Abstract

Since emotion recognition involves integration of the visual and auditory signals, it is likely that sensory impairments worsen emotion recognition. In emotion recognition, young adults can compensate for unimodal sensory degradations if the other modality is intact. However, most sensory impairments occur in the elderly population and it is unknown whether older adults are similarly capable of compensating for signal degradations. As a step towards studying potential effects of real sensory impairments, this study examined how degraded signals affect emotion recognition in older adults with normal hearing and vision. The degradations were designed to approximate some aspects of sensory impairments. Besides emotion recognition accuracy, we recorded eye movements to capture perceptual strategies for emotion recognition. Overall, older adults were as good as younger adults at integrating auditory and visual information and at compensating for degraded signals. However, accuracy was lower overall for older adults, indicating that aging leads to a general decrease in emotion recognition. In addition to decreased accuracy, older adults showed smaller adaptations of perceptual strategies in response to video degradations. Concluding, this study showed that emotion recognition declines with age, but that integration and compensation abilities are retained. In addition, we speculate that the reduced ability of older adults to adapt their perceptual strategies may be related to the increased time it takes them to direct their attention to scene aspects that are relatively far away from fixation.

## Introduction

A fundamental component of human communication is speech, but to correctly perceive the underlying message the speaker's emotional intent also needs to be correctly perceived and recognized. Emotion recognition in daily life involves optimal integration of the visual and auditory signals conveyed by the speaker. Sensory impairments could thus impair emotion recognition, although it is also possible that any remaining intact senses can, at least partially, compensate for an impaired sense. However, as sensory impairments occur relatively often in older individuals (see, e.g., Fischer et al., 2009), it is unknown whether general aging or age-related cognitive decline confounds the effects of sensory impairments on emotion recognition, or whether older age could possibly increase the negative effects of sensory impairments by limiting compensatory abilities. As a step towards studying the effects of sensory impairments on emotion recognition in individuals with dual sensory impairments, here we examined the role of stimulus degradations on recognition accuracy and perceptual strategies for emotion recognition in older adults with normal hearing and vision. Additionally, we compared these with previous findings in younger adults with normal hearing and vision (de Boer et al., 2021).

### Sensory Impairments and Their Effects on Emotion Recognition

The most common permanent sensory impairments in the older population that may impact emotion recognition are age-related hearing loss (affecting up to half of the elderly population; Fischer et al., 2009; Roets-Merken et al., 2014a; Roth et al., 2011), which is generally a sensorineural hearing loss with decreased auditory sensitivity in higher frequencies (Gates & Mills, 2005; Roth et al., 2011), and age-related macular degeneration (AMD), where a deterioration of the macula leads to central vision loss (affecting up to twenty percent of the elderly population; Colijn et al., 2017; Wong et al., 2014). While cataract is technically more common than AMD (Steinmetz et al., 2021), cataract can be treated quite well and generally does not lead to permanent vision loss. Both age-related hearing loss and AMD can be expected to impact emotion recognition, because of the loss of auditory emotion cues and difficulty of seeing face details clearly, respectively. For hearing loss, it has been shown that both children and older individuals with hearing loss show poorer auditory emotion recognition than normal hearing controls (Christensen et al., 2019; Most & Aviner, 2009; Nagels et al., 2020; Rigo & Lieberman, 1989). While hearing aids were shown to improve emotion recognition marginally, they do not seem to restore emotion recognition to the levels of normal hearing younger or older listeners (Goy et al., 2016). Additionally, individuals with AMD show poorer facial emotion recognition than controls (Boucart et al., 2008; Johnson et al., 2017), and this difference remained even when the stimulus was magnified up to twice its original size. In addition, eye movements of AMD individuals were much more variable in position than eye movements of controls (Johnson et al., 2017).

However, it is unclear whether existing findings in individuals with unimodal sensory impairments are mostly due to the effect of degraded sensory input, or confounded by a general aging effect, a long-term adaptation to the impairment, or cognitive decline brought about by aging or the impairments. For example, Orbelo et al. (2005) found that elderly participants (between 65 and 83 years of age) that had mild hearing loss and did not wear hearing aids showed decreased auditory emotion recognition. However, this decrease could not be explained by their hearing loss, nor by age-related cognitive decline, leading the authors to conclude that the decrease was related to a general aging effect. It should be noted that in this study, the participants had pure-tone hearing thresholds of on average 24 dB HL (±12 dB, average of both ears) only. Consequently, the participants’ hearing loss may have been too mild to measurably affect their performance.

In addition to the possible confounding effect of age, studies on the effects of unimodal sensory impairments on emotion recognition give little to no insight about the possible consequences of dual sensory impairments for emotion recognition, which can occur relatively frequently, that is, up to thirty percent, in the older population (Fischer et al., 2009; Guthrie et al., 2016; Saunders & Echt, 2007; Schneider et al., 2011). Therefore, as a first step, in a previous study (de Boer et al., 2021), we established the individual and combined effects of audio and video degradations on emotion recognition in a healthy group of young volunteers. By means of stimulus degradations, we intended to approximate some of the purely sensory and instantaneous consequences of hearing and vision impairments in simulation, that is, the lack of sensory input only, and not long-term adaptation or cognitive changes that may occur in real sensory impairments. The audio and video signals were degraded to mimic a moderate age-related sensorineural hearing loss and a relative central scotoma (i.e., reduced sensitivity within the scotomatic region, but not a complete loss of perception), respectively. We found that isolated audio and video degradations, that is, presenting degraded audio or video without presenting the corresponding other sense, decreased emotion recognition performance to a similar degree. However, while presenting degraded video alongside normal audio decreased performance, presenting degraded audio alongside normal video did not affect performance. Moreover, degrading both the audio and the video led to a similar performance decrease as only degrading the video. Thus, for dynamic video stimuli at least, the isolated effects of degradation do not necessarily get exacerbated when combined. Moreover, intact vision may compensate for degraded audio, but intact audio cannot compensate for degraded vision. In addition, as evidenced by eye-tracking, we found that participants adapted their perceptual strategies, by making larger saccades and looking away from the face of the actor, in response to video degradations, but not to audio degradations. These adaptations may compensate to a certain degree for the visual degradation, but this is unknown.

### The Effects of Age on Emotion Recognition

What remains unclear is whether this compensation also occurs in older adults, especially considering that there is evidence from previous research for a global decline in emotion recognition with aging (see Gonçalves et al., 2018; Ruffman et al., 2008 for meta-analyses). It has been proposed that audiovisual emotion recognition peaks between 15 and 30 years of age and declines linearly after that (Olderbak et al., 2019). Despite this general decline, there is some evidence that older adults benefit more from multimodal stimuli than younger adults such that no age-related deficits could be established in the multimodal conditions (Hunter et al., 2010; Wieck & Kunzmann, 2017). However, others found that older adults show a similar benefit to younger adults from multimodal stimulus presentation, such that the age-related difference observed in unimodal stimuli is preserved for multimodal stimuli (Lambrecht et al., 2012). In addition to a possible increased benefit from multimodal stimuli, there is some evidence for preserved or even superior emotion recognition in older adults for positive emotions, especially happiness, the so-called “positivity effect” (Calder et al., 2003; Moraitou et al., 2013; Orgeta & Phillips, 2007; West et al., 2012), at least for the recognition of facial expressions. The positivity effect is proposed to arise from an attentional bias towards positive and away from negative emotions (Carstensen & DeLiema, 2018; Mather & Carstensen, 2005). In summary, it is thus unclear whether an age-related deficit in emotion recognition will be found when using multimodal stimuli, especially for displays of positive emotions.

Besides changes in emotion recognition ability across the lifespan, there is also evidence from eye-tracking studies that older adults view emotional expressions differently from young adults. Studies have found that older adults tend to focus more on the mouth or bottom half of the face than on the eyes or top half, whereas young adults show a reversed tendency (Sullivan et al., 2007; Wong et al., 2005). In the study by Wong et al. (2005), looking at the bottom half of the face was negatively correlated with recognition accuracy for the emotions of anger, fear, and sadness, providing a straightforward explanation for why older adults may have impaired recognition of negative emotions.

### The Current Study

Real-life emotion recognition almost always involves dynamic stimuli (i.e., during face-to-face conversations) and both younger and older adults seem to recognize dynamic emotion stimuli somewhat better than static emotion stimuli (Blais et al., 2017; Khosdelazad et al., 2020). Therefore, aiming for good ecological validity, in our present study we presented dynamic stimuli, in the form of short movie-clips, to healthy young and older adults. These stimuli and the applied stimulus degradations were the same as in our previous study (de Boer et al., 2021). The use of these simulations creates a homogeneous fictitious “patient” group, while the use of two age groups allows disentangling the effects of (simulated) hearing and vision impairment from general aging effects. We used eye-tracking to examine perceptual strategies (i.e., determine when observers look where and what kind of eye-movements they make to achieve this), especially important here as some studies have shown that older adults view emotional expressions differently than younger adults.

Based on existing literature, we expected that older participants would have worse performance on emotion recognition than young participants (Gonçalves et al., 2018; Ruffman et al., 2008). However, for intact audiovisual stimulus conditions, older adults might recognize the expressed emotions with the same accuracy as younger adults, owing to previous work showing that audiovisual integration provides a larger benefit for older adults (Hunter et al., 2010; Wieck & Kunzmann, 2017). Additionally, we expected that older adults would perform as well as, or even better than young adults for positive emotions, both in intact audiovisual and unimodal conditions (Calder et al., 2003; Moraitou et al., 2013; Orgeta & Phillips, 2007; West et al., 2012). Finally, in our previous work (de Boer et al., 2021) with young normal hearing adults, we found evidence for compensation, as degraded audio did not reduce performance if it was accompanied by any video, regardless of whether the video was intact or degraded. As cognitive functioning declines with age (for a review, see Deary et al., 2009), we expected that older adults might not compensate for degraded information as well as young adults do.

Since gaze allocation is a flexible information-seeking process (de Boer et al., 2020; Hayhoe & Ballard, 2005; Võ et al., 2012), we expected that gaze patterns would differ between conditions as well as between age groups. In line with previous findings (Sullivan et al., 2007; Wong et al., 2005), for all conditions we expected that older adults would fixate more on the lower facial features (specifically, the mouth) than on the upper facial features (the eyes) compared to younger adults. Additionally, older adults’ gaze adaptations to degradations could either be similar to those of young adults or these would be less adaptive or even entirely different from those of young adults. Finally, in line with our hypothesis that age effects in performance would be neutralized in multimodal conditions, we expected that this would also hold for gaze such that older participants would attend more to the upper facial features in the multimodal conditions compared to the unimodal conditions.

## Methods

In the present experiment, both performance and eye-tracking data were obtained to identify accuracy of emotion recognition and gaze patterns during emotion perception with dynamic stimuli, respectively. The methods, including stimuli, procedures, and analyses used in this study closely resemble those used in previous studies by the authors (de Boer et al., 2020, 2021). The original—unmodified—stimulus materials were first described in (Bänziger et al., 2012).

In the study by de Boer et al. (2021), emotion recognition performance and gaze behavior were studied in young, healthy observers that viewed the stimuli in three modalities: with audio and video combined, only the video, or only the audio. Their study aimed at understanding basic aspects of audiovisual integration under sensory degradations. The data collected in our present study in healthy older adults is compared to their data (de Boer et al., 2021). Lastly, for an informal comparison, preliminary data from five individuals with macular degeneration and hearing loss (called patient participants from here on) are included here.

### Participants

Twenty-four healthy, native Dutch participants, selected to be over 60 years old and self-reported to have normal vision (or corrected-to-normal vision) and normal hearing, volunteered to take part in the experiment (12 males, mean age  =  66 years, *SD*  =  3.2, range: 61–72). All participants were given sufficient information about the nature of the experiment, but were otherwise naïve as to the exact purpose of the study. Two participants did not complete the experiment because their glasses proved incompatible with the eye-tracker. One participant did not complete the experiment because the need to be in the headrest for the eye-tracking measurements made the participant uncomfortable. Therefore, a total of 21 participants completed the entire experiment (10 males, mean age  =  66 years, *SD*  =  3.4, range: 61–72).

In addition to the data collected here, a previously collected dataset for a different study with similar methods (de Boer et al., 2021) containing data from 24 young, healthy, and native Dutch participants (nine males, mean age  =  23 years, *SD*  =  2.9, range: 19–29) was used as a control dataset in the present study to test for aging effects.

Written informed consent was obtained prior to screening and data collection. The study was carried out in accordance to the Declaration of Helsinki and was approved by the local medical ethics committee (ABR nr: NL60379.042.17). All participants received a payment of €8.00 per hour for their participation.

### Screening

Participants’ eyesight and hearing were tested before the experiment. Normal visual functioning was assessed with measurements of visual acuity and contrast sensitivity (CS), using the Freiburg Acuity and Visual Contrast Test (FrACT, version 3.9.8; Bach, 1996, 2006). Normal vision was considered as a visual acuity (VA) of at least 0.80 and a logCS of at least 1.80 (corresponding to a luminance difference of ∼1% between target and surround). Visual tests were performed binocularly and on the same computer and screen as used in the main experiment, with participants wearing their regular glasses or contact lenses. Auditory functioning was assessed by measuring auditory thresholds for pure tones at audiometric test frequencies between 125 Hz and 8 kHz. Auditory thresholds were determined using a staircase method based on typical clinical procedures. The participant sat inside a soundproof booth during audiometric testing and testing was conducted on each ear, always starting with the right ear. Since some hearing loss is nearly unavoidable in older populations (Roth et al., 2011), we have used a somewhat relaxed criterion for normal hearing compared to typical clinical procedures. For older participants, we aimed for the normal hearing definition from the European Working Group on Genetics of Hearing Impairment (HI) (Martini, 1996), where the pure-tone average (PTA; the average sensitivity at 500 Hz, 1 kHz, 2 kHz, and 4 kHz) is to be as good as or better than 20 dB HL at the better ear.

**Figure 1.**
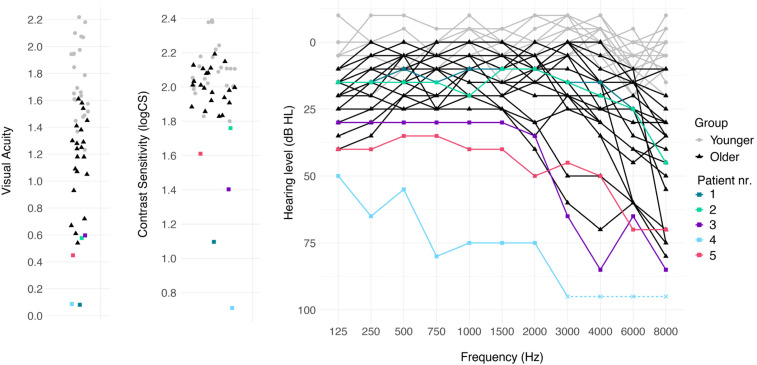
Individual levels of visual acuity (left) and contrast sensitivity (middle, in logCS), measured binocularly, for younger, older, and patient participants. Left: individual hearing thresholds in dB HL for the better ear for younger, older, and patient participants. Note: one patient participant (4) did not respond when the frequencies  ≥ 3,000 Hz were presented at 90 dB HL, at which point testing stopped to not further damage hearing. The thresholds in the figure were set at 95 dB HL to indicate this, the actual hearing thresholds for those audiometric test frequencies are unknown.

Four older participants did not have normal vision and five older participants did not have normal hearing according to our criteria (i.e., visual acuity  ≤ 0.8 and/or PTA ≥ 20 dB HL). Two participants had both non-normal vision and non-normal hearing. As a result, in total, despite perceiving themselves as normal seeing and normal hearing, seven participants did not have normal vision and/or hearing according to the criteria listed above. We still opted to keep these participants in the experiment to maintain a good number of participants. Additionally, given that they self-reported to have normal vision and hearing, these participants could still be considered representative of the aimed age group. Visual acuity, contrast sensitivity levels, and audiometric thresholds for all participants are shown in Figure 1, and individual visual acuity, contrast sensitivity, and PTA's are displayed in Supplementa1 Table A1.

Besides hearing and vision, cognitive functioning of healthy older participants was screened for using the Montreal Cognitive Assessment (MoCA). All included participants scored at or above the cut-off for normal cognitive functioning (26 points). Additional exclusion criteria were neurological or psychiatric disorders, dyslexia, and the use of medication that could influence normal brain functioning.

### Stimuli

Audiovisual emotional expressions taken from the Geneva Multimodal Emotion Portrayals (GEMEP) core set (for a detailed description, see Bänziger et al., 2012) were used as stimuli during the experiment. A short demo showing only the face of the actor can be found at the Geneva Emotion Recognition Test (GERT) demo at https://www.unige.ch/cisa/emotional-competence/home/exploring-your-ec/. The GEMEP core set consists of 145 audiovisual video recordings (mean duration: 2.5 s, range: 1–7 s) of emotional expressions portrayed by 10 professional French-speaking Swiss actors (five females) of different ages (mean: 37.1 years, range: 25–57 years). The lexical content of the expressions was one of two pseudo-speech sentences with no semantic content, but resembling the phonetic sounds in western languages (“nekal ibam soud molen!” and “koun se mina lod belam?”). Out of the 17 emotions in GEMEP, 12 were selected for the main experiment, such that they would be equally distributed over the quadrants of the valence-arousal scale. See Table 1 for the 12 emotions and how they are distributed over the valence-arousal scale (Russell, 1980). Portrayals from two actors that were found to be less clearly recognizable in previous work (de Boer et al., 2020) were used during practice trials to familiarize participants with the stimulus materials and the task. Thus, a total of 96 unique stimuli were used in the main experiment and a total of 24 unique stimuli in the practice trials.

**Table 1.** The Selected Emotion Categories. The Emotions are Evenly Distributed Over the Quadrants of the Valence-Arousal Scale (Russell, 1980).

		Valence	
		Positive	Negative
Arousal	High	Amusement Joy Pride	Fear Despair Anger
	Low	Pleasure Relief Interest	Irritation Anxiety Sadness

### Visual Stimulus Degradation

**Figure 2.**
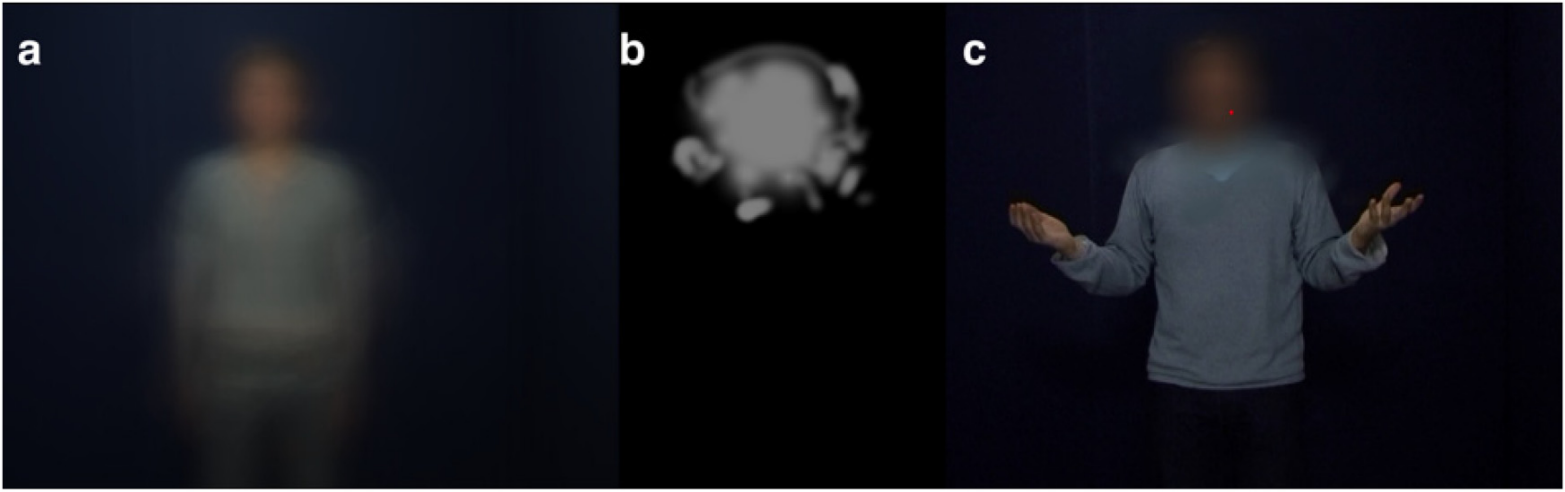
(a) Still image created by averaging together all frames of all videos. This image preceded stimulus presentation in all conditions with video. (b) Shape and approximate size of the scotoma mask. The scotoma was gaze-contingent with its center positioned on the point of gaze. Four different orientations were used during the experiment (randomly intermixed): as shown in this figure, left-right flipped, up-down flipped, and left-right and up-down flipped. (c) Scotoma overlaid on a still image of one video. The red dot indicates the point of gaze, this dot was not visible to participants. The still image in (c) is retrieved from one of the video's of the GEMEP core set from Bänziger et al. (2012). Published with permission from the Swiss Center for Affective Sciences.

A gaze-contingent relative scotoma was produced using custom MATLAB scripts. A semi-circular, yet irregular, shape that was centered on gaze position, was used to mimic the estimated vision loss in an individual with progressed binocular AMD, see Figure 2b and c. The shape of the scotoma was not based on an actual scotoma, but based on the fact that the macula spans a roughly circular region in central vision. However, as the vision loss of an individual with AMD will hardly ever be perfectly circular, an irregular shape was used. The scotoma was shown in one of four different orientations in each trial: original (as shown in Figure 2b), horizontally flipped, vertically flipped, and both horizontally and vertically flipped. Orientation was randomized between trials. The scotoma's size was roughly 17° × 11.5° visual angle (VA; 731  × 497 pixels) and had soft edges. Most AMD individuals do not perceive a hole in the location of their visual field defect, but distortions or blur (Taylor et al., 2018). Because of this, we decided to blur rather than remove the region of the video that the scotoma covered. A Gaussian low-pass filter (using the MATLAB function *fspecial* and *imfilter*), was used to create a blurred version of the video. The filter had a cut-off frequency (at full width at half maximum, FWHM) of 0.15 cycles/deg. Then, this filtered version was overlaid on the original—unfiltered—video, and the alpha-layer of the scotoma image (see Figure 2b) served to indicate what region of the video should be hazy and how strongly.

Participants were informed that the scotoma was gaze-contingent and that they could use compensatory eye-movements in order to peripherally view regions in the video they found relevant. Participants were informed that looking away from the actor could help them in still seeing the expressed emotion on the video, but were informed neither on the direction nor on the size of the eye-movements they should make in order to do so.

### Auditory Stimulus Degradation

Degradation of the audio signal was done using customized MATLAB scripts aimed at approximating three characteristics of sensorineural HI: increased absolute thresholds, loudness recruitment, and the effects of broader auditory filters on narrowband envelopes in the auditory system. The processing used here was inspired by the HI simulation of Nejime and Moore (1997). The audio manipulation consisted of two sequential modules: one for envelope processing, and one for loudness perception. The envelope module was designed to produce perceptual effects of broader auditory filters (i.e., impaired frequency resolution), while the loudness module simulated raised audiometric thresholds and loudness recruitment.

The envelope-processing module created narrowband envelopes as they are assumed to be present in the impaired auditory system via broader auditory filtering, while the fine structure should be preserved as in normal hearing. Therefore, the input audio signal was processed with a Gammatone filter bank with bandwidths of two equivalent rectangular bandwidths (ERBs), representing impaired auditory filters, at one ERB distance across center frequencies between 80 Hz and 10 kHz. The filter bandwidth of two ERB was selected as representative for moderate sensorineural HI (Moore, 1998). Within each frequency band the envelope was extracted using the Hilbert transform, which each served as the target HI envelope. Hilbert envelopes from broader filters were then multiplied onto Hilbert fine structure signals in each frequency band. Normal narrowband envelopes can be partially recovered from a NH fine structure signal by NH listeners (cf., Ghitza, 2001). To minimize this unwanted recovery of envelopes, thus to provide “degraded envelopes” inside the normal auditory system of the participants in this study, an iterative procedure was used whereby the output of the multiplication procedure was passed through the NH filter bank again and the fine structure extracted using the Hilbert transform was multiplied again by the target impaired envelopes. Ten such iterations were used in the present study, resulting in a high average correlation coefficient of 0.83 with the desired HI envelopes after modeled NH auditory processing using speech as a signal (Bennett & Hohmann, 2012).

After the envelope processing module, the loudness module sets the sound level in each frequency band such that the NH participants listening to this simulation had a similar loudness perception as an (average) HI listener. For this manipulation, the output signal of the envelope-processing module was fast Fourier transformed (FFT-ed) into six octave-spaced channels with frequencies between 250 Hz and 8 kHz. The sound level in each channel was extracted from the output signal and the categorical loudness ratings as used in the procedure of Brand & Hohman (Brand & Hohmann, 2002) were calculated based on average HI categorical loudness data (Oetting et al., 2016), which served as target loudness. The sound levels were then attenuated in an expansive fashion such that (average) NH listeners’ loudness perception of the sound level matches the target HI loudness. Finally, the spectral signal was transformed back into the time domain using the inverse FFT. The loudness module thus also set the simulated audiometric thresholds. For the present study the degradations were implemented by taking the thresholds from a moderate HI, similar to the standard audiogram N3 as defined in Bisgaard et al. (2010). The thresholds were 40, 40, 45, 54, 62, and 70 dB HL at audiometric frequencies of 250, 500, 1,000, 2,000, 4,000, and 8,000 Hz, respectively.

After these two modules, the sound level of the final output signal was root-mean-square (RMS) equalized to the intact audio, to ensure any effects found were not a side-effect of an overall decrease in presentation level.

### Experimental set-up

The experiment was performed in a dark and quiet room, with the monitor providing the only illumination. Participants sat in front of the monitor at a viewing distance of 70 cm with their head placed in a chin- and forehead rest to minimize head movements. Stimuli were presented full-screen on a 24.5-inch monitor with a resolution of 1920  × 1080 pixels (43°  × 24.8°). The average screen luminance was 38 cd/m^2^, measured from the approximate head location of the participant. An Apple MacBook Pro (mid 2015 model) was connected to the monitor and controlled the stimulus presentation. The audio was produced by the internal soundcard of this computer and presented binaurally through Sennheiser HD 600 over-ear headphones (Sennheiser Electronic GmbH & Co. KG, Wedemark, Germany). The sound level was calibrated to be at a comfortable and audible level, at a long-term RMS average of 65 dB SPL. Participants used an external mouse for responding. Stimulus display and response recording was controlled using the Psychophysics Toolbox (Version 3; Brainard, 1997; Kleiner et al., 2007; Pelli, 1997) and Eyelink Toolbox (Cornelissen et al., 2002) extensions of MATLAB (Version R2015b; The Mathworks, Inc., Natick, MA, USA).

Participant's eye movements were measured with an Eyelink 1000 Plus eye-tracker (SR Research Ltd., Ottawa, Ontario, Canada), running software version 4.51. Monocular gaze data was acquired at a sampling frequency of 1000 Hz. The eye-tracker was located just below the monitor. A calibration procedure preceded the experiment using the built-in 9-point calibration routine. Calibration accuracy was verified with the validation procedure in which the same nine points were displayed again. The experiment would start if the calibration accuracy was sufficient (i.e., average error of less than 0.5° and a maximum error of less than 1°). Drift was checked for after every fourth trial and after each break. The calibration procedure was repeated if the participant moved during breaks and whenever there was more than 1° of drift in more than one consecutive drift check.

### Procedure

During the experiment, participants were asked to identify the emotions expressed in the GEMEP core set videos. The videos were presented in eight different stimulus presentation conditions, listed in Table 2. Participants were asked to respond as accurately as possible in a forced-choice discrimination paradigm. Participants were further requested to blink as little as possible during the trial and maintain careful attention to the stimuli.

**Table 2.** Experimental Conditions Used in the Experiment. Both Modalities Were Either Shown as They are (Intact), Degraded, or Absent.

		Video		
		Intact	Degraded	Absent
Audio	Intact	AV	AdV	A
	Degraded	dAV	dAdV	dA
	Absent	V	dV	

Each trial was preceded by either a full-screen image of the averaged frames of all videos (for all conditions with video, see Figure 2a) or a fixation cross (for A and dA; conditions without video), which was presented for a random duration between 600 and 1,600 ms. For conditions with video, this averaged image was displayed instead of a fixation cross to allow participants to already orient their gaze, which could be especially beneficial in the conditions where a scotoma was present. Then, the stimulus was presented for 1–7 s, depending on the specific video. For A and dA, the fixation cross remained on screen. After stimulus presentation, a response screen appeared. On this screen, all 12 emotions were presented with a label, grouped in a circular fashion by valence and arousal. The participant could, in a forced-choice response format, click with the mouse pointer on the emotion label that corresponded to the identified emotion. All 12 emotions were always presented on the response screen. The response screen remained visible until a response was made. The participant's response (the emotion label) was recorded as well as whether the response was correct or not.

Each participant was presented with all 96 videos (12 emotions × 8 actors) in all eight conditions, each individual video was thus presented eight times. The experiment was separated into six experimental blocks and in each block, all eight conditions were presented in sub-blocks containing one sixth of the stimuli (i.e., 16 trials per sub-block, 128 trials per experimental block). The order of conditions between experimental blocks was counterbalanced using balanced Latin Squares within and across participants. For young participants, the stimulus order for each condition was fully randomized. For older participants, the stimulus order was pseudo-randomized: they saw the videos from a set of four pseudo-randomly chosen actors (two male, two female) in the first session, and the videos from the remaining four actors in the second session. Stimulus order within each set of four actors was randomized. The reason for this change was that we had expected many older participants would drop out of the study after one session due to the length of the experiment. With this change, at least we would have balanced data after one session (i.e., all emotions presented equally often in all conditions). In the end, none of the older participants dropped out for this reason.

The experiment was preceded by 64 practice trials (eight practice trials for each condition) to acquaint the participants with the stimulus material and the task. For the practice trials, all conditions were presented in the following fixed order: AV, V, A, AdV, dAV, dV, dA, dAdV. Stimulus order within each practice block was randomized. During the practice block, participants received minimal feedback after each trial on their given response (i.e., correct/incorrect). No feedback was provided during the experiment.

Overall, the experiment consisted of 832 trials, including the 64 practice trials, and took about 2.5 h to complete. The experiment was separated over two test sessions performed on separate days to avoid fatigue. Participants were able to take a self-paced break every 32 trials and were encouraged to take breaks in order to maintain concentration and prevent fatigue. The experiment continued upon a mouse-click from the participant and the eye-tracker was recalibrated if the participant moved during the break.

### Data Analyses

The data analysis was performed in two stages. The first analysis stage focused on intact conditions (A, V, and AV). The second stage focused on the effects of audio and video degradation (dA, dV, AdV, dAV, and dAdV). All data (i.e., accuracy scores, fixation durations, saccadic amplitudes, and fixation proportions) were analyzed in R (version 3.6.0; R Foundation for Statistical Computing, Vienna, Austria—https://cran.r-project.org) with linear regression models (using *lmer* from the *lme4* package, version 1.1–21). Since our main interest was in the effect of age, only the main effects of age group and interactions with age group were followed-up by post-hoc tests. Other variables (e.g., condition, emotion) were added if they improved the model. For both stages, the best model was found by comparing Akaike Information Criterion (AIC) values for the different models. The criterion for picking a more complex model was an AIC decrease of at least two (cf. Wieling et al., 2014). Significance of main effects and interactions of the final models were assessed with an Analysis of Deviance table (type III Wald chi-square test) with the *Anova* function from the *car* package (version 3.0–3). Significant effects were followed up by post-hoc tests to test how age groups differ. Post-hoc tests were performed using *lsmeans* from the *emmeans* package (version 1.4.1). Note that many of our analyses were exploratory, meaning that we did not have clear hypotheses a priori for these analyses (especially concerning the effect of different conditions for both age groups). In those cases, the corrections for multiple comparisons were generally not strict, and some of the findings may not survive more stringent corrections.

#### Analyses of Behavioral Data

Accuracy scores for each condition and emotion were first converted to unbiased hit-rates (de Boer et al., 2020, 2021; Wagner, 1993) to account for any response biases. The unbiased hit-rate (*H_u_*) is different from the regular hit-rate in that it also considers false alarms. It can be calculated by squaring the number of correct responses for a category, and dividing that by the number of occurrences of that category times the number of times this particular response was used. In our study, an example of the *H_u_* for the emotion Joy would be the Joy_correct_ ^2^/(Joy_occurrence_*Joy_responded_). Because of this, if a participant often responds to Joy correctly (i.e., Joy_correct_ is high), but this is due to a bias towards responding Joy (i.e., Joy_responded_ is higher than Joy_occurrence_), the unbiased hit-rate will be lower than the regular hit-rate to account for this bias. The unbiased hit-rates were arcsine transformed (Sokal & Rohlf, 1995) to create a normal distribution. Then, a linear regression analysis was performed with the arcsine transformed *H_u_* as the dependent variable.

In both stages, the base model included the condition (with three/eight levels), age group (young/old), and their interaction as fixed effects. Then, participant was included as a random intercept and emotion was included in steps (i.e., first as random intercept, then as main effect, then in interaction with age group and/or condition), making the model more complex with each step. Additionally, we tested whether the inclusion of random slopes for condition and/or emotion improved the model. As mentioned, the AIC was used to test whether the model improved with the added complexity and in addition, if the more complex model did not converge, the addition was excluded. Post-hoc tests were performed using Bonferroni correction for multiple comparisons in the first stage, and with the false discovery rate (FDR) correction for multiple comparisons in the second stage.

#### Analyses of eye-tracking data

For the eye-tracking data, the built-in data-parsing algorithm of the Eyelink eye-tracker was used to extract fixations from the raw eye-tracking data. Only data from conditions in which the video was present (all except A and dA) were analyzed, as in the conditions without video participants would have mostly been fixating on the fixation cross throughout the trial. All analyses were restricted to eye movements made during stimulus presentation, and only those made within 1,000 ms after stimulus onset. No gaze data after 1,000 ms were considered to limit data analysis to the duration of the shortest movie, which lasted 1,000 ms. In addition, this aimed to discard any data that no longer was task-related, that is, after a participant decided on a response, which is increasingly likely to occur at a longer interval after stimulus onset. Trials with single blink longer than 300 ms during the first 1,000 ms of stimulus presentation were discarded. Additionally, only trials with a correct response were included, as our main interest was in gaze behavior prior to correct recognition. Focusing on correct responses allowed examining whether changes in gaze behavior due to information degradation and availability of audio were adaptive and lead to good performance.

Mixed linear regressions were performed to test for the effects of age group, condition, emotion on fixation durations and saccadic amplitudes. For fixation proportions, AOI was included as an additional fixed effect. Random intercepts were included for participant and movie and random slopes for condition were included if they improved the model.

Fixation durations and saccadic amplitudes were extracted from the parsed data file. Saccades with amplitudes larger than the diagonal of the monitor, which was 49.6°, were filtered out, removing less than 1% of saccades. An exploratory mixed linear regression was performed for both fixation duration and saccadic amplitude.

Additionally, we performed an area-of-Interest (AOI) based analysis on fixations for those conditions in which the video was present. For fixation proportions on AOIs, the eyes (left and right), nose, mouth, and hands (left and right) of the actors were chosen as AOIs. Because the stimuli are dynamic, the AOIs were dynamic as well. Coordinates of the AOI positions for each stimulus and each frame were extracted using Adobe® After Effects® (Version 15.1.1; Adobe Inc., San Jose, CA, USA). The coordinates for the face AOIs were obtained by applying the “Face Tracking (Detailed Features)” method of Adobe® After Effects®, which automatically tracks many face features. Face track points at each frame were visually inspected and manually edited whenever the tracking software failed to track them correctly. For the hand AOIs, the “Track Motion” method of Adobe® After Effects® was used. A single tracker point per hand was used to track position. The tracker point was placed roughly in the center of the hand. Again, tracking was inspected visually and manually edited where needed (for more details on face and hand tracking, see de Boer et al., 2020). Coordinates of all obtained face and hand track points for each stimulus were stored in text-files and used to create point AOIs. For the eyes we used the coordinates of the “Left/Right Eyebrow Outer” for the *x*-position of the lateral corner, “Left/Right Eyebrow Inner” for the *x*-position of the medial corner, “Left/Right Eyebrow Middle” for the top, and the middle between the *y*-positions of “Left Pupil” and “Nose tip” for the bottom, indicating the eye–nose border. The individual AOIs for the left and right eye were later merged for analysis. For the nose we used the eye–nose border as the top, the nose–mouth border as the bottom (middle between the *y*-positions of “Right Nostril” and “Mouth Top”), the *x*-position of “Right Nostril” and the *x*-position of “Left Nostril” for the lateral corners. For the mouth AOI: the *x*-position of “Mouth Right” and the *x*-position of “Mouth Left” for the lateral corners, the nose–mouth border for the top, and the *y*-position of “Mouth Bottom” for the bottom. Each AOI was expanded by 10 pixels on each side (20 pixels across the horizontal and vertical axes), except at the eye–nose and nose–mouth borders. Overlap between AOIs was avoided. The actual size of each AOI varied across actors and frames, for example, due to some actors being closer to the camera. Note that left and right are in reference to the actor, not the observer. Thus, the left eye and hand are generally on the right side of the screen and vice versa for the right eye and hand.

Fixation proportions on the AOIs were defined as follows: for all of the *N* fixation time-points, the fixation proportion is the proportion of *N* that is located on a given AOI. These proportions were then averaged over each trial, resulting in a mean fixation proportion on each AOI for each trial. These means were finally arcsine-transformed. A mixed linear regression was performed on the arcsine-transformed mean proportions.

### Data From Patient Participants

We collected data from five individuals (two males, mean age  =  69, SD  =  4.44, range: 66–77) with some form of macular degeneration and, for three cases, also some hearing loss. All patient participants were screened in the same way the healthy younger and older participants were screened. Unlike in the healthy older participants, the MoCA was not administered in patient participants because their vision and hearing loss may negatively affect the outcome and lead to the spurious conclusion that their cognitive functioning is poorer. In addition, standard automated perimetry (HFA Central 10-2 protocol) was obtained and all filled in the Dutch versions of the Speech and Spatial Qualities (SSQ 5.6, home version) and the Visual Functioning Questionnaire (VFQ-25/NL, home version) to assess how they experience their hearing and vision impairments. HFA results are included in Supplemental Figure A1, questionnaire outcomes are summarized in Supplemental Tables A2 and A3.

For patient participants, the general set-up was the same as for healthy participants; each patient participant was presented with all 96 stimuli in sub-blocks of 16 trials. However, only the A, V, and AV conditions were used, which in principle should correspond to the dA, dV, and dAdV conditions because of the patient's vision and HIs. The experiment was thus also preceded by only 24 practice trials (eight practice trials for each condition) in which the conditions were shown in the following order: AV, V, and A. In total, the experiment for the patient participants consisted of 312 trials, including the 24 practice trials and took about 1.5 h to complete. The experiment was completed in one session. We also collected eye-tracking data from the patient participants, but calibrating the eye-tracker properly proved impossible due to their central visual field defect. Therefore, the eye-tracking data from the patient participants was too noisy to properly analyze and we only describe patient participants’ emotion recognition accuracy results.

## Results

### Age Effects on Accuracy for Intact Conditions

**Figure 3.**
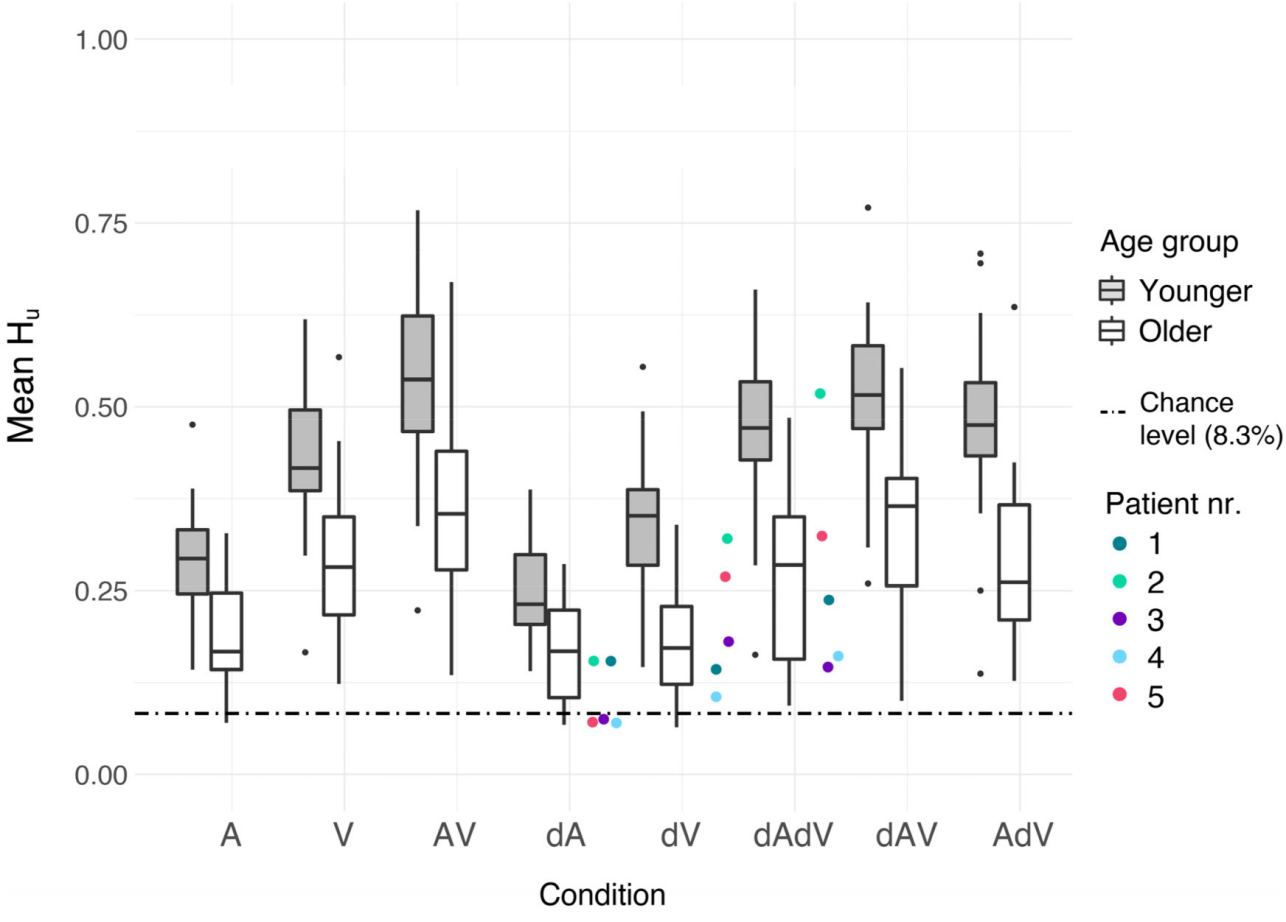
Task performance for each condition and age group, shown as unbiased hit-rates. Performance is averaged across emotions and blocks. Each box shows the data between the first and third quartiles. The horizontal solid line in each box denotes the median. The whiskers extend to the lowest/highest value still within 1.5*inter-quartile range (IQR), data outside the 1.5*IQR are plotted as dots. Performance for young participants is shown in light grey boxes, performance for older participants is shown in white boxes. Performance for individual patient participants is shown in the colored dots. Note that these participants did not receive degraded stimuli, but their hearing and visual acuity tests indicate that their perception is degraded. Thus, for patient participants, dA corresponds to stimuli presented in A, likewise for dV and dAdV. The dashed line indicates chance level performance.

Emotion recognition performance is shown in unbiased hit-rates in Figure 3. Please note that while analyses were performed on the arcsine transformed *H_u_*, Figure 3 plots non-transformed *H_u_* for interpretability. Figure 3 shows that, overall, the performance (quantified as unbiased hit rates) of older participants was lower than that of the younger ones. It also appears that, for both age groups, performance was lowest in A, intermediate in V, and best in AV. The best regression model (i.e., the most complex model with the lowest AIC value) to test this included *condition (only A, V, and AV)* in interaction with *age group*, *condition* in interaction with *emotion*, and *emotion* in interaction with *age group*. *Participant* was included as random intercept, with a random slope for *condition*. Thus, the formula for the final model was: H_u_asin_ ∼ condition*age  + condition*emotion +  emotion*age  +  (condition|participant). All main effects were significant (all *p* < .001). Additionally, the interactions between *condition* and *emotion* (*Chi^2^* [22]  =  117.9, *p* < .001) and between *age group* and *emotion* (*Chi^2^* [11]  =  37.4, *p* < .001) were significant. The interaction between *age group* and *condition* was not significant (*Chi^2^* [2]  =  5.3, *p*  =  .07).

Follow-up post-hoc tests on the main effect of *condition* confirmed that performance was the lowest for A, intermediate at V, and best for AV (all *p* < .001). The significant main effect of *age group* confirmed that older participants performed poorer than younger participants (difference estimate  =  0.17, *t*  =  4.64, *p* < .001). Older participants performed significantly poorer than young participants for all emotions, except for the emotions Joy (difference estimate  =  0.08, *t*  =  1.52, *p*  =  .13) and Anxiety (difference estimate  =  0.09, *t*  =  1.91, *p*  =  .06), even though the latter differences were in the same direction as for the other emotions.

### Age Effects on Accuracy for Degraded Conditions

To investigate the effects of degradations, a regression model with *condition (all conditions)* in interaction with *age group*, *condition* in interaction with *emotion*, and *emotion* in interaction with *age group* was performed. *Participant* was included as a random intercept, but without a random slope for *condition*, as this led to a singular fit. Thus, the formula for the final model was: H_u_asin_ ∼ condition*age  +  condition*emotion  +  emotion*age  +  (1|participant) All main effects were significant (all *p* < .001). Additionally, there were significant interactions between *condition* and *emotion* (*Chi^2^* [77]  =  393.7, *p* < .001), between *age group* and *emotion* (*Chi^2^* [11]  =  108.5, *p* < .001), and between *age group* and *condition* (*Chi^2^* [7]  =  42.9, *p* < .001).

Follow-up post-hoc tests showed that older participants had lower accuracy for all conditions (all *p* < .002) and all emotions (all *p* < .009), including positive ones. The significant interaction between *age group* and *emotion* indicates that the differences between younger and older participants were not the same for all emotions. Additionally, while the patterns across conditions appeared very similar for both age groups, there were subtle differences, see Table 3. For instance, degrading video seemed to reduce performance more in older than in younger participants. Note that Table 3 only lists sensible comparisons, for example, A is compared to dA, but not to dV.

**Table 3.** Contrasts for the Age Group by Condition Interaction for Recognition Accuracy.

Comparison	Age group	
	Younger	Older
A – dA	**0.06** **(** **0.001)**	**0.05** (**0.008)**
V – dV	**0.09** (**<0.001)**	**0.16** (**<0.001)**
AV – dAdV	**0.08** (**<0.001)**	**0.12** (**<0.001)**
AV – dAV	0.02 (0.337)	0.03 (0.109)
AV – AdV	**0.06** (**<0.001)**	**0.09** (**<0.001)**
dAdV – dAV	**−0.07** (**<0.001)**	**−0.09** (**<0.001)**
dAdV – AdV	−0.02 (0.180)	−0.03 (0.109)

The table shows the model estimate differences with the false discovery rate (FDR) adjusted *p*-values in parentheses. Significant differences are indicated by bold typeface.

Additionally, Figure 3 shows that the five patient participants that were included had a similar emotion recognition accuracy as the included older healthy participants had in the degraded A, V, and AV conditions. These preliminary data support the idea age-related sensory changes can affect audiovisual emotion recognition, and our degradations captured some of these effects in individuals with no sensory impairments.

### Effects of Auditory and Visual Functioning on Emotion Recognition Accuracy

Overall, older participants had poorer hearing and vision than the younger participants, even though the older participants perceived themselves as having normal hearing and vision. This was tested by a two-sample *t*-test (function *t.test* from the R *stats* package, version 4.0.3), equal variances not assumed. The differences between younger and older participants were significant for all screening outcomes: PTA (*t*[26.1]  =  −6.86, *p* < .001, mean_younger_  =  0.89, mean_older_  =  14.46), visual acuity (*t*[39.9]  =  6.67, *p* < .001, mean_younger_  =  1.75, mean_older_  =  1.16), and contrast sensitivity (*t*[41.2]  =  2.55, *p*  =  .015, mean_younger_  =  2.10, mean_older_  =  2.0). Because of these differences, an additional model was constructed that included PTA, visual acuity (VA), and contrast sensitivity (CS): H_u_asin_ ∼ condition*age  +  condition*emotion  +  emotion*age  +  PTA  +  VA  +  CS  +  (1|participant). However, the effects of *PTA* (*Chi^2^* [1]  =  0.46, *p*  =  .50), *VA* (*Chi^2^* [1]  =  0.06, *p*  =  .81), and *CS* (*Chi^2^* [1]  =  1.16, *p*  =  .28) were not significant while the effect of *age* (*Chi^2^* [1] =  5.48, *p*  =  .02) was still significant, indicating that the poorer hearing and vision of the older participants seemed not to be the reason for their lower emotion recognition accuracy.

### Age Effects on Fixation Duration for Intact Conditions

**Figure 4.**
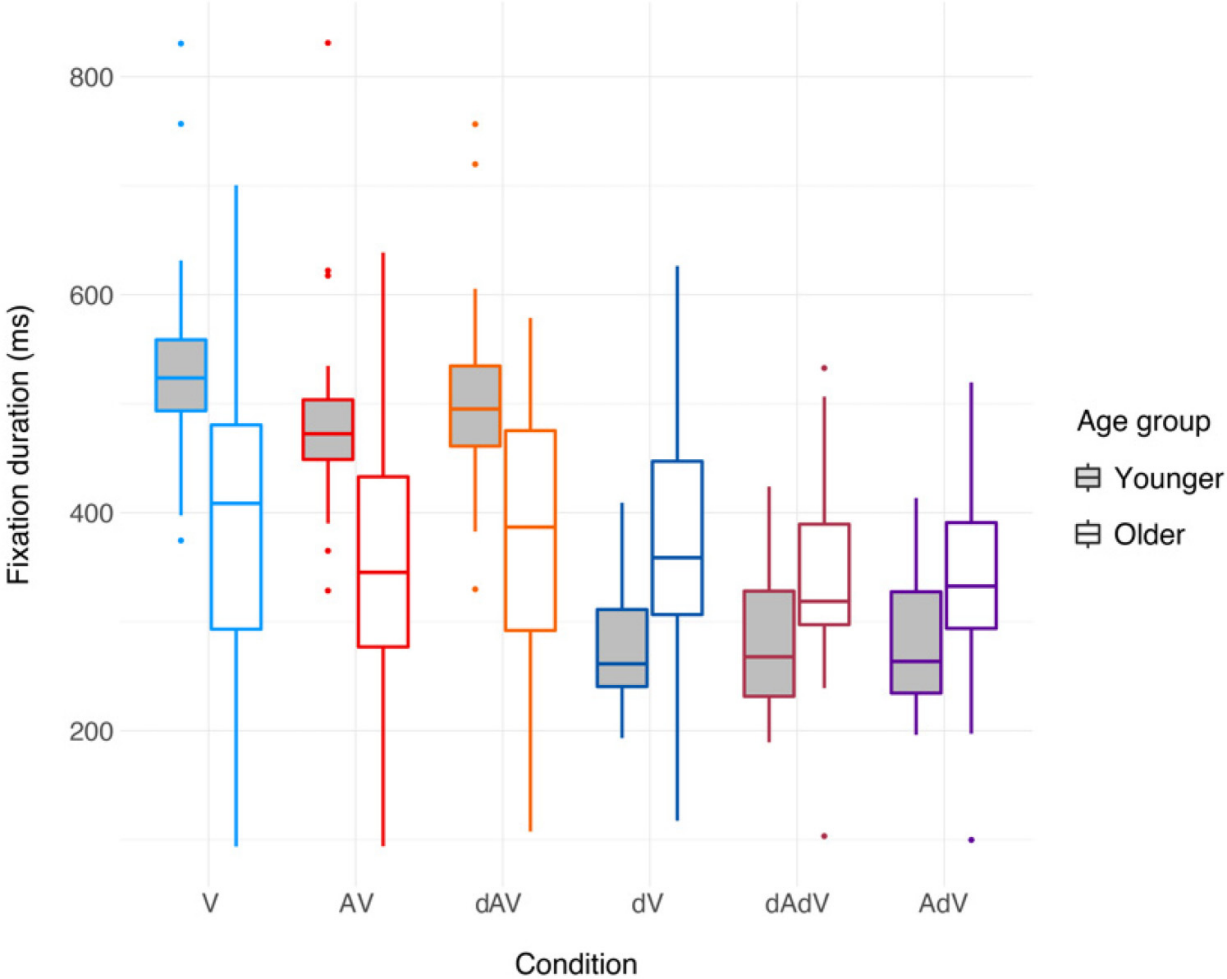
Fixation durations in ms for all conditions and age groups. As for Figure 3, fixation durations are averaged across emotions and blocks. Each box shows the data between the first and third quartiles. The horizontal solid line in each box denotes the median. The whiskers extend to the lowest/highest value still within 1.5*IQR, data outside the 1.5*IQR are plotted as dots. Performance for young participants is shown in light grey boxes, performance for older participants is shown in white boxes.

Figure 4 shows that, on average, older participants tended to have shorter fixation durations than younger participants. In addition, there seems to be a small effect of condition.

The regression models confirmed this. The best model included *condition* and *age group* as main effects only, a random intercept for *participant*, with a random slope for *condition*, and a random intercept for *movie*. The formula for the final model was: duration ∼ condition  +  age  +  (condition|participant)  +  (1|movie).

The main effects of *condition* (*Chi^2^* [1]  =  27.6, *p* < .001) and of *age group* (*Chi^2^* [1]  =  12.3, *p* < .001) were significant. A follow-up of these main effects showed that fixations were of longer duration in the V compared to the AV condition (difference estimate  =  53.2, *t*  =  5.25, *p* < .001). Additionally, older participants made fixations of shorter duration than younger participants (difference estimate  =  126, *t*  =  3.43, *p*  = .001).

### Age Effects on Fixation Duration for Degraded Conditions

From Figure 4, it can be seen that younger participants adapt their gaze to the degraded video by making fixations with a shorter duration. Older participants do not seem to show the same adaptation, or they do so to a smaller degree. The best model to test this included *age group* and *condition* as main effects as well as their interaction. Random intercepts were included for *participant* and *movie*, but without any random slopes as these led to a singular fit. Thus, the formula for the final model was: duration ∼ condition*age  +  (1|participant)  +  (1|movie). Both the main effect of *age group* (*Chi^2^* [1]  =  18.0, *p* < .001) and of *condition* (*Chi^2^* [5]  =  2243.3, *p* < .001) were significant, as well as the interaction between *condition* and *age group* (*Chi^2^* [5]  =  599.1, *p* < .001).

Post-hoc tests of the interaction between *condition* and *age group* showed that, in general, participants decreased fixation duration in conditions with degraded video. However, the differences were much smaller for older participants than for younger participants. For younger participants, the decrease in mean fixation durations with degraded video compared to intact video was significant (all *p* < .001) and on average 225 ms, while for older participants the average decrease was significant in most cases (*p* < .013), except for the comparisons between AV and dV (*p*  =  .507) and dAV and dV (*p*  =  .340), but was only 11 ms. There even appeared to be a small increase in fixation duration when comparing AV and dV in older participants, although this difference was not significant (difference estimate  =  −7.6, *p*  =  .507). For both groups, fixation durations were longest in the V condition and fixation duration did not differ between AV and dAV.

### Age Effects on Saccadic Amplitude for Intact Conditions

**Figure 5.**
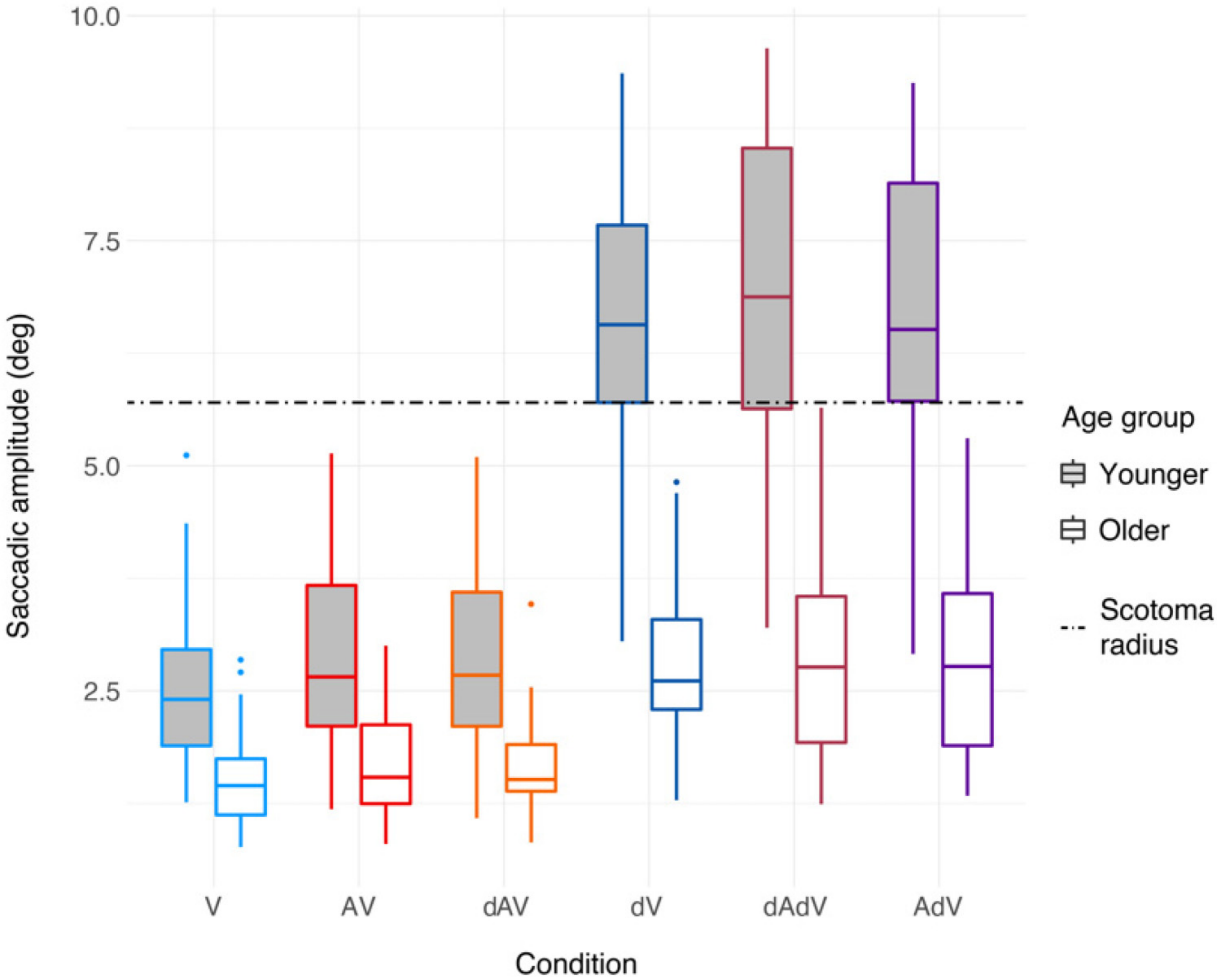
Saccadic amplitudes in degree of visual angle for all conditions and age groups. Amplitudes are averaged across emotions and blocks. Each box shows the data between the first and third quartiles. The horizontal solid line in each box denotes the median. The whiskers extend to the lowest/highest value still within 1.5*IQR, data outside the 1.5*IQR are plotted as dots. Performance for young participants is shown in light grey boxes, performance for older participants is shown in white boxes. The dashed line indicates the minimal radius of the scotoma.

Figure 5 shows that older adults generally made saccades with a smaller amplitude than younger adults. The regression models confirmed this. The best model included *condition* and *age group* as main effects only, a random intercept for *participant*, with a random slope for *condition*, and a random intercept for *movie*. The formula for the final model was: amplitude ∼ condition  +  age  +  (condition|participant)  +  (1|movie).

The main effects of *condition* (*Chi^2^* [1] =  13.0, *p* < .001) and of *age group* (*Chi^2^* [1]  =  15.9, *p* < .001) were significant. A follow-up of these main effects showed that saccades were larger in the AV compared to the V condition (difference estimate  =  0.24, *t*  =  3.60, *p* < .001). In addition, older participants made smaller saccades than younger participants (difference estimate  =  1.01, *t*  =  3.91, *p* < .001).

### Age Effects on Saccadic Amplitudes for Degraded Conditions

From Figure 5, a similar result to what was observed for fixation duration, is seen for saccadic amplitudes. Participants adapt their gaze to degraded video by making larger saccades in those conditions, but older participants seem to make smaller adjustments than younger ones.

The regression model confirmed this. The best model included *condition* and *age group* as main effects as well as their interaction. Random intercepts were included for *participant* and *movie*, but without any random slopes as these led to a singular fit. The formula for the final model was: amplitude ∼ condition*age  +  (1|participant)  +  (1|movie). Both the main effects of *condition* (*Chi^2^* [5]  =  4713.4, *p* < .001) and *age group* (*Chi^2^* [1]  =  12.6, *p* < .001), as well as the interaction (*Chi^2^* [5]  =  889.2, *p* < .001) were significant.

The follow-up post-hoc comparisons had results similar to those for fixation duration. All participants adapted their gaze to degraded video by making larger saccades, although the differences were smaller for older participants. For younger participants, the increase in saccadic amplitudes for degraded video conditions was on average 3.70° (from 2.83° in intact video conditions to 6.54° in degraded video conditions), while for older participants the increase was only 1.20° (from 1.58° in intact video conditions to 2.78° in degraded video conditions). The increases in saccadic amplitudes were significant for all comparisons between degraded and intact video conditions and for both age groups (all *p* < .001) Additionally, only younger participants made significantly smaller saccades in V compared to the AV and dAV conditions (AV – V  =  0.29, *p*  =  .005; dAV – V  =  0.24, *p*  =  .015). For older participants, there was a trend in the same direction (AV – V  =  0.16, *p*  =  .225; dAV – V  =  0.17, *p*  =  .225).

### Age Effects on Fixation Proportions for Intact Conditions

**Figure 6.**
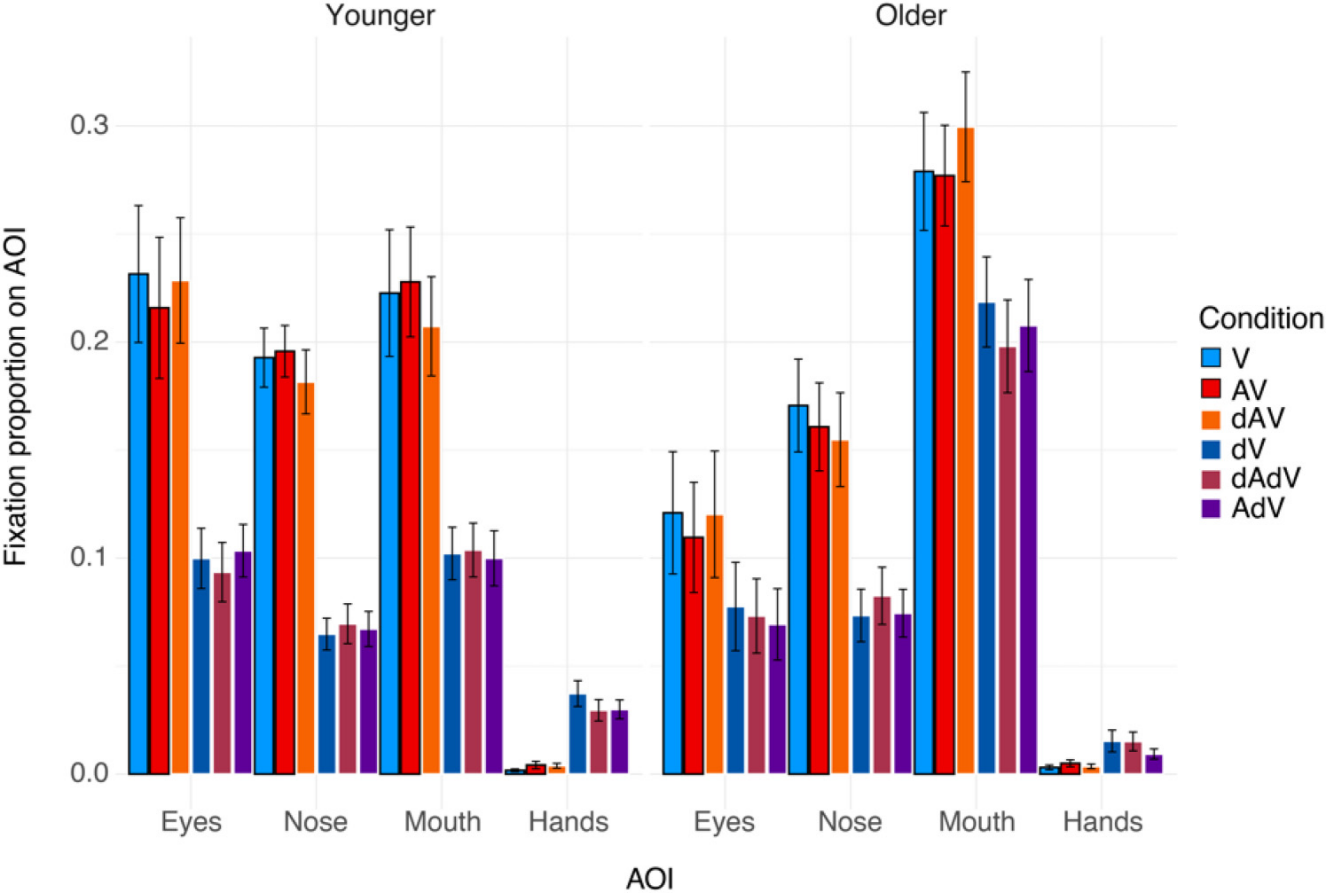
Mean fixation proportions on the face and hand AOIs (areas of interest) for all conditions and both age groups, and averaged over emotions and blocks. Error bars denote the standard error of the mean (SEM). Intact conditions are indicated by a black outline.

Figure 6 shows that all participants fixate more on the face than on the hands of the actors. Additionally, it appears that younger participants distribute their fixations more or less equally across the face AOIs, but that older participants focus mostly on the mouth.

The final model included *age group* in interaction with *AOI*, and *AOI* in interaction with *emotion*. Random intercepts were added for *participant* and *movie*, but no random slopes were added as these led to a singular fit. *Condition* did not have a significant effect on fixation proportions, both as a main effect and in interaction with any of the other variables (all *p* > .33) and was therefore taken out of the final model. Thus, the formula for the final model was: proportion ∼ AOI*age  +  AOI*emotion  +  (1|participant)  +  (1|movie). All main effects were significant (all *p* < .001), as well as the interaction between *age group* and *AOI* (*Chi^2^* [3]  =  263.3, *p* < .001) and between *AOI* and *emotion* (*Chi^2^* [33]  =  122.4, *p* < .001).

A follow-up of the interaction between *age group* and *AOI*, using an FDR-corrected post-hoc test, showed that older participants fixated more often on the mouth than younger participants (difference estimate  =  0.08, *t*  =  4.33, *p* < .001), but less often on the eyes (difference estimate  =  0.16, *t*  =  8.97, *p* < .001).

### Age Effects on Fixation Proportions for Degraded Conditions

Fixation proportions for all conditions and both age groups are shown in Figure 6. For both age groups, participants fixated less on the face AOIs in conditions with degraded video. Additionally, the bias for older participants to fixate more on the mouth was also present for the dAV condition, perhaps even stronger, and remained present under degraded video.

The best regression model included main effects of *AOI*, *age group*, *condition*, and *emotion*, as well as interactions between *AOI*, *age group*, and *condition*, and between *AOI* and *emotion*. Thus, the final model formula was: proportion ∼ AOI*age*condition  +  AOI*emotion  +  (1|participant)  +  (1|movie). All main effects were significant (all *p* < .012). Additionally, there were significant interactions between *age group* and *AOI* (*Chi^2^* [3]  =  10.0, *p*  =  .018), between *AOI* and *Condition* (*Chi^2^* [15]  =  1023.8, *p* < .001), *AOI* and *emotion* (*Chi^2^* [33]  =  59.1, *p*  =  .003), and between *age group*, *AOI*, and *condition* (*Chi^2^* [15]  =  130.6, *p* < .001). Because our main interest was in age effects, only the interactions between *age group* and *AOI*, and between *age group*, *AOI*, and *condition* were followed-up with post-hoc tests.

The *age group* by *AOI* interaction showed that, overall, young participants fixated significantly more often on the face AOI than the hands (all *p* < .001), with no differences between the fixation proportions on the face AOI (all *p* > .266). Conversely, while older participants also fixated more on the face than on the hands (all *p* < .001), they additionally fixated more on the mouth than on both the nose (difference estimate  =  0.19, *t*  =  5.40, *p* < .001) and the eyes (difference estimate  =  0.25, *t*  =  4.22, *p* < .001). All comparisons for the *age group* by *AOI* by *condition* interaction are shown in Supplemental Table A4. In general, all participants fixate less on the face AOIs in degraded video conditions, and young participants additionally fixate more on the hands in those conditions. The differences were generally smaller for older than for younger participants. Lastly, young participants fixated less on the mouth for the dAV condition compared to AV (with a similar trend for dAV compared to V), but older participants fixated more on the mouth for the dAV condition compared to both AV and V.

In summary, results from the first analysis stage showed that older participants had lower accuracy scores than younger participants, but older participants were as capable of integrating auditory and visual information as younger participants were. There was no evidence for a “positivity effect” for older participants, as their accuracy was lower for all emotions. Additionally, older participants made smaller saccades and fixations of shorter durations than younger participants. Lastly, older participants fixated mostly on the mouth of the actor, while younger ones distributed their fixations roughly equally over the actors’ face.

From the second analysis stage, we found that, for both age groups, audio degradation did not reduce performance if the degraded audio was accompanied by intact video. Moreover, presenting degraded audio and degraded video simultaneously did not reduce performance more than only degrading the video and leaving the audio intact. Lastly, older participants did not adapt their gaze behavior as much as young participants.

## Discussion

Our main finding is that older participants were as good as younger participants at integrating audio and video during the recognition of emotions presented using the AV stimulus materials. Likewise, both groups were equally good at compensating for degraded audio. However, in contrast to these comparable relative effects, older participants were systematically poorer at recognizing emotions than younger adults. Their recognition accuracy was lower in all conditions and for nearly all emotions compared to that of the younger participants. This age effect could not be explained by a difference in visual and auditory functioning. Both age groups had a higher accuracy in the video-only than in the audio-only conditions, and accuracy was highest during AV presentation. Notably, the differences in performance between these conditions were similar for both age groups. Additionally, degrading the video always reduced recognition accuracy, regardless of whether the degraded video was presented in isolation or together with audio, while degraded audio only reduced accuracy when it was presented in isolation. This suggests that participants rely more strongly on the visual than on the auditory information when judging emotions with these stimulus materials.

In addition to these differences in recognition accuracy, we found that older participants had a strong fixation bias towards the mouth of the actor, while young participants distributed their fixations more evenly across the face. When presented with the video degradations, younger participants made much larger saccades, presumably in an attempt to move the scotoma away from the face and view the face with their peripheral vision. While older participants did so too, their increase in saccadic amplitude was much smaller. Consequently, their saccades were not large enough to move the scotoma away from the face. Our results thus confirm that emotion recognition deteriorates with age and we additionally show that age also affects gaze behavior.

Lastly, even though we have not formally analyzed the data from the patient participants due to the small sample size, their data still provide some useful preliminary insights. In general, the patient participants performed similarly as the older healthy participant group did in the degraded conditions, with both groups being of similar age. This similarity is an indication that our stimulus degradations captured at least some of the consequences of actual hearing and vision loss on emotion recognition. However, individual differences in performance between patient participants were very large, and were presumably at least partly related to their vision and hearing loss. For example, patient participant 2 had relatively good visual acuity (0.58) and contrast sensitivity (1.76 logCS), relatively little visual field loss, and only some hearing loss in higher frequencies (and normal PTA: 16.3 dB HL). In all conditions, this patient participant had the highest accuracy. In contrast, patient participant 4 had both poor visual acuity (0.09) and contrast sensitivity (0.71 logCS), had much more visual field loss, and was completely deaf in one ear and had severe hearing loss in the other ear (PTA: 68.3 dB HL), and this patient participant had very low accuracy in all conditions. Perceived auditory and visual functioning, measured with the SSQ and VFQ-25 respectively, were loosely correlated with the results from the screening. Although other factors, such as age, education level, and how long they have had impaired vision and hearing likely also contribute to differences in emotion recognition accuracy across patient participants, it appears that differences in visual field loss, visual acuity, contrast sensitivity, and hearing levels at least partially explain the individual performance differences.

### Older and Younger Adults Integrate Audiovisual Information for Emotion Recognition Similarly

For all intact conditions (A, V, AV) and all emotions, older participants showed lower emotion recognition accuracy than young participants. This is in line with other findings (see Gonçalves et al., 2018; Ruffman et al., 2008 for meta-analyses). Additionally, we found that the addition of another modality did not change the accuracy difference between older and younger participants that was observed for unimodal modalities. Rather, when only considering the intact conditions, there was no significant age group by condition interaction, indicating that the difference in accuracy remained roughly the same across A, V, and AV conditions. Therefore, unlike what has been previously reported (Hunter et al., 2010; Wieck & Kunzmann, 2017), we find that older participants are as good as younger participants at integrating auditory and visual information, but not better. Wieck and Kunzmann (2017) already proposed that divergent findings could be due to differences in the quality of the emotion expression. They hypothesized that older adults only benefit from additional information (in other modalities) if that additional information clearly points towards the same emotion. In our experiment, due to the large number of different emotions included, the emotional cues in each modality may have been subtler and more complex than in previous studies, such that integrating auditory and visual cues does not necessarily resolve all ambiguity. The chance of that happening is much smaller when there are fewer emotions being portrayed; Wieck and Kunzmann (2017) only presented two emotions (anger and sadness), and Hunter et al., presented four (fear, sadness, disgust, and anger). In our study, in contrast, 12 emotions (of which six were negative) were used, and some were closely related (e.g., anger and irritation). We consider our approach a more ecologically valid approximation of real life, in which people do not always display their emotions very consistently and clearly, do not limit themselves to core emotions only but instead display a wide range of emotions. Therefore, we claim that our results are a relatively good representation of emotion recognition abilities in daily life, and the earlier studies may not have been sufficiently sensitive as a result of using too few emotion categories.

It is worth noting that the difference in accuracy between younger and older participants is not (fully) driven by poorer vision and hearing in the older group, as shown by our analysis in the section ‘Effects of Auditory and Visual Functioning on Emotion Recognition Accuracy’.

### The Ability to Compensate for Sensory Degradation Remains Stable With Age

For both age groups, we found that our signal degradations decreased recognition accuracy. When presented in isolation (i.e., unimodal degraded stimulus presentation), degraded audio/video (dA, dV) led to lower accuracy than for unimodal intact audio/video stimulus presentation (A, V). Besides this, older participants showed roughly the same pattern across degraded conditions as younger participants did: degraded video combined with intact (AdV) or degraded audio (dAdV) led to a similar decrease in accuracy compared to AV. Only degrading audio (dAV), however, did not lead to a decrease in accuracy compared to AV. Therefore, it seems that, at least for the task and materials used here, participants could fully compensate for the degraded audio by relying more on the visual information. In contrast, relying more on intact auditory information to compensate for degraded video was not possible. Moreover, these effects were the same for both the younger and older participants. This similarity suggests that, although emotion recognition ability may decline with age, the ability to compensate for sensory degradation seems to remain stable with advance age.

### No Evidence for a Positivity Effect, but an Overall Emotion Recognition Reduction With Age

We found that older adults’ recognition accuracy was poorer compared to young participants’ accuracy for both positive and negative emotions. There was therefore no evidence for a positivity effect in our data, contradicting some previous findings (Calder et al., 2003; Moraitou et al., 2013; Orgeta & Phillips, 2007; West et al., 2012). Again, this discrepancy with literature could be related to the large number of emotions that were used in the current study. The task of discriminating between many different emotions, and additionally integrating auditory and visual information, which were sometimes degraded, likely lead to a high cognitive load. There is evidence that high cognitive load reduces or completely diminishes the positivity effect (Knight et al., 2007; Noh & Isaacowitz, 2015). Additionally, previous findings of a positivity effect may have been related to the fact that these studies used little positive emotions. All these studies (Calder et al., 2003; Moraitou et al., 2013; Orgeta & Phillips, 2007; West et al., 2012) only used the six basic emotions (happiness, surprise, sadness, fear, anger, disgust). Only two emotions of the six basic emotions are positive, and only happiness is very clearly positive, while surprise is a bit more ambiguous. Therefore, the reason that these studies find that recognition of positive emotions is preserved with age, may be solely due to the fact that it is easier to correctly guess the positive emotions if there are only two positive emotions in the stimulus set.

### Older Adults Tend to Fixate More on the Mouth, While Younger Adults Distribute Fixations Evenly Across the Face

For intact conditions, older participants had a strong tendency to fixate on the mouth of the actor, which is in line with previous findings (Sullivan et al., 2007; Wong et al., 2005). This bias towards fixating on the mouth was traded off by a decrease in fixations on the eyes. Younger participants, however, distributed their fixations more evenly over the actor's face. Both age groups hardly ever fixated on the hands of the actor. The bias of older adults to fixate on the mouth (or at least, bottom half of the face) more has been indicated to be related to their preserved ability for recognizing positive emotions (Wong et al., 2005), as a prototypical expression of happiness is most clearly recognizable by the smiling mouth (Bassili, 1979; Calder et al., 2000). However, here we showed that while older adults generally have this bias, the accuracy difference between younger and older adults still remains for positive emotions. Therefore, it remains to be examined why this bias exists in older adults. Contrary to our hypothesis, the fixation bias towards the mouth remained in multimodal conditions, but this is line with the finding that the age effect for performance also remained in multimodal conditions. In addition to the difference in fixation proportions, older participants on average had shorter fixation durations and additionally made smaller saccades.

### Reduced Gaze Adaptation in Older Adults

All participants adapted their gaze to degraded video presentation (dV, AdV, dAdV), but did not adapt their gaze in response to degraded audio (dAV), for which there were no significant differences with AV. For both age groups the gaze adaptations to degraded video were apparent as a decrease in fixation durations, an increase in saccadic amplitudes, and a decrease in fixation proportions on all face AOIs. However, these changes were much smaller for older participants than they were for younger participants. For example, younger participants increased saccadic amplitudes from on average 2.5° of visual angle in intact video conditions (V, AV, dAV) to about 6° for degraded video conditions (dV, AdV, dAdV). In contrast, older participants showed saccadic amplitudes of on average 1.5° for intact video conditions, and increased to on average 2.5° for degraded video conditions. Since the scotoma extended 17 by 11.5°, making saccades of 6°, as the young participants generally did, would be sufficient to move the scotoma away from the face.

These results suggest that there was a limitation in older adults’ vision, eye movements, or cognitive processing that makes it impossible or less optimal to make the large gaze adaptations that younger adults do, although it is uncertain what exactly. One possible explanation is that older adults consistently make hypometric saccades, and because of this never “reach” the target with their gaze. However, several studies on the effects of age on saccade dynamics do not show an effect on saccadic amplitude or accuracy (Mack et al., 2020; Pratt et al., 2006; Warabi et al., 1984), making this an unlikely explanation for our findings. A potentially straightforward explanation is that within the relatively short time span of fixation, older observers are not capable of attending to items that are far away from their point of gaze. Indeed, it has been shown that when given the same amount of time to inspect a display, older adults have a narrower spatial spread of attention compared to younger adults (Lawrence et al., 2018) and a smaller useful field of view (i.e., the visual area in which useful information can be acquired within a brief timespan; Coeckelbergh et al., 2004; Sekuler et al., 2000). Therefore, we propose that within the typical duration of their fixations, the older participants in our study were incapable of attending to the face if it was far out in their visual periphery and therefore optimized their performance by fixating closer to it.

Note that we only analyzed trials with correct recognition, as we assumed that this would inform on whether the adapted gaze behavior would lead to good performance. However, an extra analysis (not included here) showed that there was no difference in gaze behavior for incorrect versus correct recognition for both age groups. Based on this, it can be concluded that observers settle on a gaze adaptation strategy (consciously or unconsciously) that optimizes performance as much as the restrictions of that participant's visual and cognitive systems allow.

### Limitations and Future Directions

Our findings, especially those related to the fact that visual information seems more important than auditory information, may be strongly dependent on the specific materials used here. The video stimuli had very rich visual cues, including both facial expressions and body language, and possibly less clear auditory cues, which only included prosodic but not semantic information. Therefore, future studies should test the assumption that vision can compensate for degraded audition (be it simulated or real) by using different audiovisual emotion materials, for example by including sentences with meaningful semantic content.

In addition, we cannot rule out that our results were not driven by differences in other factors that have been indicated to impact emotion recognition processes, such as education level (see Gonçalves et al., 2018), cultural differences, and cognitive functioning (e.g., Phillips et al., 2008). While the present study confirmed with the MoCA that none of our older participants showed signs of cognitive impairment, we did not directly assess cognitive functioning in both groups. Likewise, we did not assess participants’ education level and as most of the younger participants were university students, it is possible that there was a difference in education level between the younger and older participant groups.

Lastly, the fact that we analyzed eye-movements over a relatively short time period of 1,000 ms, may have affected what differences we observed between age groups. For example, it is possible that older adults needed more time during the trial to start exhibiting adapted gaze behavior and a short temporal analysis window may not have captured this properly. However, as mentioned in the methods section (see Data Analyses—Analyses of eye-tracking data), the time period was chosen to fit the length of the shortest video and to ensure that only task-related gaze data was included. It may be worthwhile to study this by using emotion stimuli that morph from neutral to emotional over different time spans and study whether morph duration affects age differences in gaze.

### Conclusions

Altogether, the present data show that audiovisual integration for emotion recognition remains intact with age, even though aging seems to lead to a general decrease in emotion recognition abilities. Additionally, we have shown that both younger and older adults adapt their perceptual strategies in response to degraded visual information, although older adults make smaller adaptations than younger adults. These smaller adaptations may be related to the smaller useful field of view in older adults. Therefore, rehabilitation programs aimed at expanding the useful field of view (see Edwards et al., 2018) and teaching adapted viewing behavior to visually impaired individuals may improve their emotion recognition. However, before implementing this, further studies into the mechanisms and benefits of gaze adaptation are necessary.
